# In Vitro Proliferation of MG-63 Cells in Additively Manufactured Ti-6Al-4V Biomimetic Lattice Structures with Varying Strut Geometry and Porosity

**DOI:** 10.3390/ma17184608

**Published:** 2024-09-20

**Authors:** Dimitri P. Papazoglou, Laura Hobbs, Yvonne Sun, Amy Neidhard-Doll

**Affiliations:** 1 Department of Electrical and Computer Engineering, University of Dayton, Dayton, OH 45469, USA; aneidharddoll1@udayton.edu; 2 Department of Biology, University of Dayton, Dayton, OH 45469, USAysun02@udayton.edu (Y.S.)

**Keywords:** lattice structure, additive manufacturing, Ti-6Al-4V, MG-63, osteogenesis

## Abstract

Lattice structures have demonstrated the ability to provide secondary stability in orthopedic implants by promoting internal bone growth. In response to the growing prevalence of lattices in orthopedic design, we investigated the effects of porosity and unit cell geometry in additively manufactured Ti-6Al-4V biomimetic lattice structures on the osteogenesis of human MG-63 osteoblastic cell lines in vitro. We analyzed glucose consumption, alkaline phosphatase (ALP) concentration, and end-of-culture cell count as markers for osteogenic growth. Two different strut geometries were utilized (cubic and body-centered cubic), along with four different pore sizes (400, 500, 600, and 900 µm, representing 40–90% porosity in a 10 mm cube), in addition to a solid specimen. Structural characterization was performed using scanning electron microscopy. The results indicated that lattices with a 900 µm pore size exhibited the highest glucose consumption, the greatest change in ALP activity, and the highest cell count when compared to other pore sizes. Cubic 900 µm lattice structures outperformed other specimens in facilitating the maturation of viable MG-63 cells from the formation to the mineralization phase of bone remodeling, offering the most promise for osseointegration in additively manufactured titanium implants in the future. However, irrespective of a particular pore size or unit cell geometry, it was found that all the lattices were capable of promoting osteogenic growth due to surface roughness in the printed parts.

## 1. Introduction

In recent years, there has been an increase in research focused on the design of additively manufactured lattice structures using biocompatible materials, such as Ti-6Al-4V, for orthopedic implants. Traditionally, commercial orthopedic devices (e.g., hip and knee implants) have been created through manufacturing methods such as cast-molding or multi-axis machining. While these ‘legacy’ methods are advantageous in the production of large quantities of devices in a limited number of shapes and sizes, they fail to address anatomical differences across various demographics (e.g., age, sex, height, weight, etc.), which influence physiological attributes such as bone density and biomechanical loading. In addition, these ‘legacy’ methods cannot produce complex 3D geometries such as lattice structures that are utilized in customized solutions (e.g., synthetic scaffolds used to repair bone fractures).

Recent technological advances in selective laser melting (SLM) additive manufacturing technology (e.g., in situ process monitoring) have afforded researchers the opportunity to explore 3D-printed orthopedic implants for in vivo use. In 2017, the United States Food and Drug Administration (FDA) published technical considerations for additively manufactured medical devices [1] and has since granted 510 k clearance to various lumbar interbody fusion devices that feature 3D-printed lattice structures produced by SLM [2,3]. The development of patient-specific implants for reconstructive applications involving unique deformities or injuries has also demonstrated the promise of additive manufacturing in the medical setting [4,5,6]. For example, Stoffelen et al. [4] developed a glenoid implant using computed tomography (CT) data to 3D print a titanium implant that matched the patient’s anatomy for optimal fixation. Two and a half years after surgery, the patient’s Shoulder Pain and Disability Index decreased from 72% (pre-surgery) to 28% (post-surgery); in addition, the implant showed no signs of loosening. Wong et al. [6] designed a patient-specific pelvic implant using patient CT and MRI data to create a 3D model of the bone tumor. Based upon the virtual bone tumor, a porous scaffold implant was designed and fabricated by SLM using Ti-6Al-4V material and inserted surgically. Ten months post-operatively, the patient was able to walk without assistance, and the implant showed satisfactory alignment with no loosening. In addition to the promise of additive manufacturing for improved patient outcomes, the use of additively manufactured orthopedic implants can improve the efficiency of the supply chain through reducing the waste of raw materials as well as reduced manufacturing time [6].

The incorporation of porous, viscoelastic lattice structures in the design of additively manufactured orthopedic implants may further enhance both mechanical and biological properties. One of the common problems associated with traditional orthopedic devices is stress shielding, in which the higher material stiffness associated with a metallic implant results in decreased mechanical loading of the residual bone [7]. Over time, stress shielding results in complications in the patient, such as bone loss, thereby reducing the recovery potential of the bone and increasing the risk of fracture [8]. Biomimetic lattice structures inspired by the highly porous (75–95%) network of interconnected rods and plates found in trabecular bone may offer a potential solution [9]. Through modifying the porosity and strut geometry (i.e., unit cell geometry) in additively manufactured lattice structures, the effective modulus of elasticity (the relationship between an applied force and the subsequent deformation response of a given material under various loading conditions) of an orthopedic implant can be customized for specific patient needs, thereby reducing stress-shielding, and improving the overall outcome for the patient.

One of the primary biological benefits of implementing lattice structures in the design of orthopedic implants is the facilitation of osteogenesis, occurring both externally and internally [4,5,6,10,11,12,13,14,15,16,17]. For a non-porous ‘traditional’ implant, the post-operative bone remodeling phenomenon (where growth of the extracellular matrix occurs only on the implant surface) leads to mature bone growth only on the implant surface [18]. In the case of porous implants, the extracellular matrix can extend internally into the body of the implant, providing a greater level of osseointegration when compared to nonporous implants. The relationship of external bone fusing with the porous network of a lattice can improve mechanical interlocking, improving the secondary stability of an implant [19,20].

The average range of pore sizes that past studies have found appropriate for osseointegration is 400–1000 µm [10,11,12,13,14,15,16,17,19,20,21,22,23,24,25,26,27,28], with some investigations indicating that pores as small as 200 µm [24] and as large as 1200 µm [22,23] can promote osteogenesis. Wally, et al. [26] studied numerous pore sizes (400, 650, 250 µm) in SLM-manufactured Ti-6Al-4V lattices, finding that all pore sizes supported cell growth and mineralized matrix deposition. The only notable difference was with pore size 400 µm, which had larger calcium depositions. Srivas, et al. [12] observed osseointegration through bone ingrowth with 500 µm pore-sized Ti-6Al-4V scaffolds. Similarly, de Wild, et al. [17] analyzed SLM-produced titanium cubic lattice structures with 500–600 µm pore size, noting internal bone cell growth in vivo for rabbits with calvaria defects.

There is also evidence to suggest that the structural geometry of lattice structures, notably the interconnectivity of pores, can influence osteogenesis. Wang, et al. [27] investigated the cell behavior of lattice structures of various strut geometries: diamond crystal lattice, regular distribution; diamond crystal lattice, irregular distribution; diamond crystal lattice, gradient distribution; and a tetrahedral structure. For all lattices, the average pore size was 500 µm and the average strut size was 400 µm. The lattices were produced by SLM using Ti-6Al-4V material. All four strut geometries demonstrated internal osteogenic growth, but no significant differences were found across any of the structures in terms of cell viability, proliferation, and morphology. Markhoff, et al. [28] observed the influence of three different lattice strut geometries on human osteoblast cells. The three structures were cubic (pore size 700 µm, strut size 700 µm), pyramidal (pore size 400–620 µm, strut size 400 µm), and diagonal (pore size 400–1000 µm, strut size 400 µm). The lattices were produced with both SLM (cubic, pyramidal, and diagonal) and Electron Beam Melting (EBM) (only cubic) using Ti-6Al-4V material. Results indicated the pyramidal structure had the highest level of cell activity and migration when compared to cubic and diagonal.

Prior noted studies have demonstrated lattices with large (300–600 µm) strut thickness. In this work, we investigated the effects of porosity and unit cell geometry in SLM additively manufactured Ti-6Al-4V biomimetic lattice structures that contain a designed strut thickness of 200 µm. We analyze osteogenesis of human MG-63 osteoblastic cell lines in vitro, using glucose consumption, alkaline phosphatase (ALP) concentration, and end-of-culture cell count as markers for osteogenic growth. Two different strut geometries were utilized (cubic and body-centered cubic), along with four different pore sizes (400, 500, 600, and 900 µm, representing 40–90% porosity in a 10 mm cube), in addition to a solid specimen for comparison.

## 2. Materials and Methods

### 2.1. 3D-Printed Ti-6Al-4V Lattice Structures

**Figure 1:**
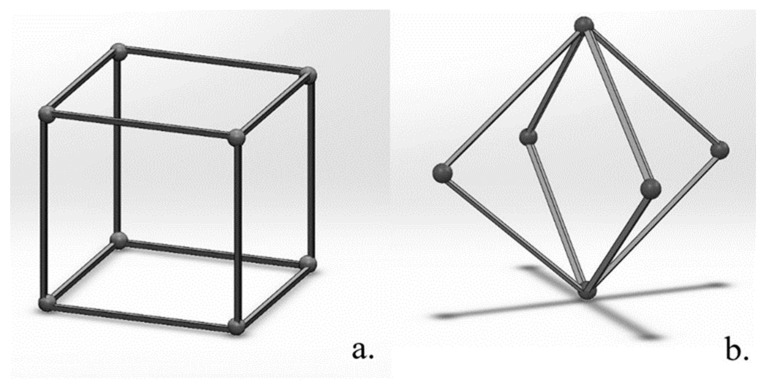
Unit cell geometry of cubic (**a**) and body-centered cubic (**b**).

**Figure 2:**
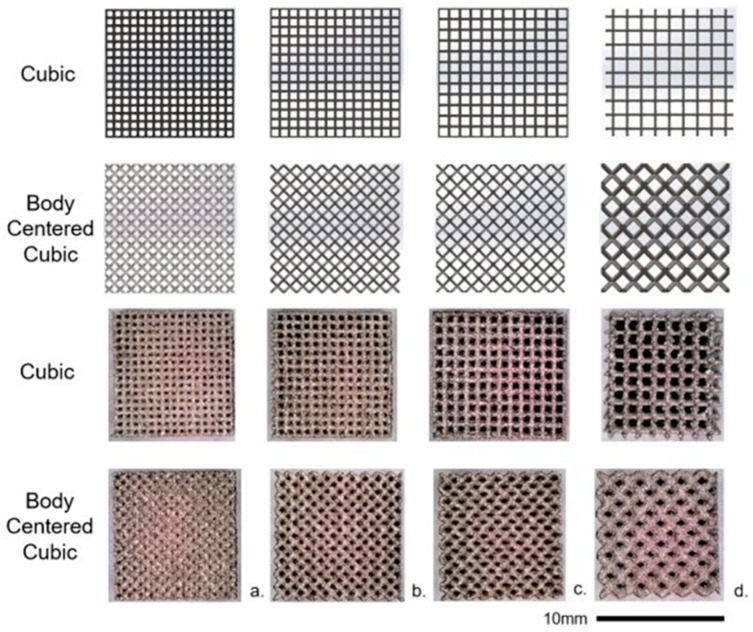
CAD visualizations (**top two rows**) and SLM as manufactured (**bottom two rows**) images of 10 mm cubic and body-centered cubic lattices. Pore sizes: (**a**) 400 µm, (**b**) 500 µm, (**c**) 600 µm, and (**d**) 900 µm.

**Figure 3:**
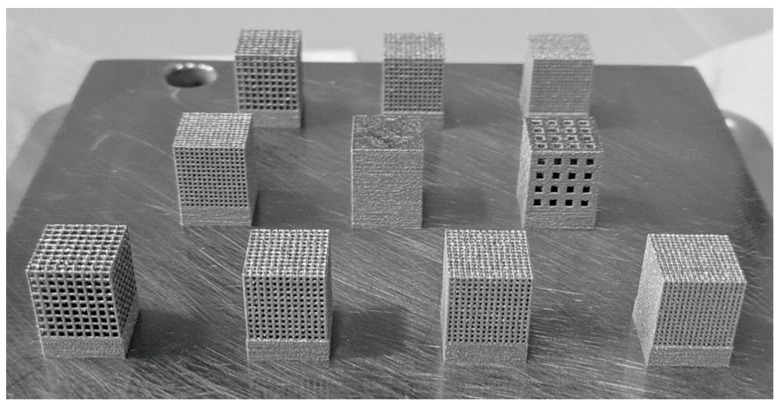
SLM build plate for Ti-6Al-4V lattices (cubic cell geometry, porosity 400–900 µm) prior to heat treatment.

Biomimetic lattice structures with cubic and body-centered cubic (BCC) unit cell geometries were designed using engineering design software nTopology version 3.0 (Figure 1). The lattice specimens designed for in vitro testing were 10 mm^3^ cubes. For each unit cell geometry (cubic and BCC), the pore size was modified (400, 500, 600, and 900 µm), for a total of eight unique lattices for the experiment (Figure 2). Cubic and BCC lattices were chosen due to their known ability to be 3D printed, along with demonstrated mechanical properties appropriate for orthopedic applications. Each lattice had a 200 µm strut thickness, with the exception of BCC 900 µm—which had a 400 µm strut thickness. The eight lattices (along with a solid 10 mm^3^ cube) were 3D printed in Ti-6Al-4V powder using an open-architecture SLM machine (DART SLM; University of Dayton Research Institute, Dayton, OH, USA) with an IPG Photonics Continuous Wave 500 W laser. During the build, laser power was set to 150 W with a mark speed of 950 mm/s and a bidirectional strip hatch with 6 mm titles and 0.08 mm spacing. Laser thickness was 40 microns. Oxygen content during the build was >0.1%. The powder was Ti-6Al-4V Grade 5 (Reuse 9). Referencing ASTM F3456, the powder used for this study followed the ‘continuous reuse’ schema [29]. The powder was sieved at 63 µm and 75 µm to remove any anomalies. Multiple builds were printed to produce replicate lattices required for cell culture experiments, with each build incorporating eight lattices of the same unit cell geometry and various pore sizes (Figure 3).

After each build was completed, the parts were heat treated on the build plate at 800 °C (±10 °C) for 120 min (±30 min) in vacuum (1.3 × 10^−3^–1.3 × 10^−5^ mbar), followed by cooling under vacuum. The lattices were removed from the build plate by electron discharge machining. To remove excess powder and disinfect the lattices, each lattice was placed in an ultrasonic cleaner with 70% ethyl alcohol for four rinses at one hour each, followed by four water rinses for one hour each. Lastly, each lattice was autoclaved for 40 min at 121 °C.

### 2.2. MG-63 Cell Culture Preparation

MG-63 human bone cells (Model CRL-1427, ATCC, Manassas, VA, USA) were grown in Dulbecco’s Modified Eagle Medium (DMEM) with 10% (*v*/*v*) heat-inactivated fetal bovine serum (FBS), 100 units/mL penicillin, 100 mg/mL streptomycin, 5 mM sodium pyruvate, and 10 mM L-glutamine. For six to nine days, MG-63 cells were grown in an incubator with 5% CO_2_ at 37 °C until approximately ~1.4 × 106 cells were available for each lattice. Cell counting was manually performed with an inverted microscope using a hemocytometer containing 10 µL of cell suspension. During the passage of the bone cells, the lattices were submerged in DMEM for one day on a shaker plate (60 RPM) for the lattices to become saturated. The next day, lattices were placed in untreated conical tubes containing ~1.4 × 106 cells per lattice and DMEM for seeding. Each conical tube was then placed on a shaker plate (60 RPM) for 24 h to allow the cells to become distributed internally within the lattices. After the seeding period, each lattice was placed into an individual well of a 24-well plate with 1.5 mL of cell culture media. The media was replaced every four days to maintain a consistent amount of free glucose available to developing MG-63 cells, as well as to eliminate cells growing outside of the lattice specimen in suspension. This technique was implemented to isolate glucose consumption by MG-63 cells that permeated the pores and/or adhered to the surface of each titanium lattice structure.

### 2.3. Cell Culture Tests

**Figure 4:**
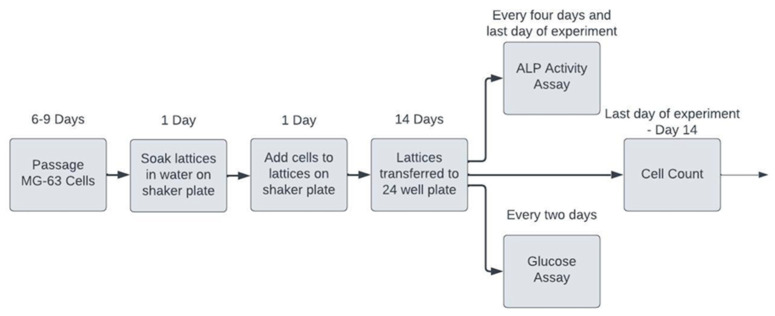
Flow chart illustrating the in vitro cell culture experiment procedures.

A total of eight unique Ti-6Al-4V lattices of varying cell geometry (cubic or BCC) and pore size (400, 500, 600, or 900 µm) were used in the in vitro cell culture experiments. One solid Ti-6Al-4V 10 mm^3^ cube was included for comparison. The experiment was repeated three times, with new lattices and solid specimens used each time to eliminate the effects of residual cell debris and contaminants from prior experiments. A flow chart illustrating the procedures followed for in vitro cell culture experimentation can be found below in Figure 4.

#### 2.3.1. Glucose Assay

The glucose assay (Catalog Number 23-666-286; Fisher Scientific, Hampton, NH, USA) was performed every two days per the manufacturer’s instructions to measure glucose concentration in the culture media as a proxy for cellular metabolic activity. Each sample (5 µL) was diluted 1:2 with phosphate buffered saline (PBS) prior to the assay to ensure sample glucose concentrations within the linear range of the assay.

#### 2.3.2. ALP Assay

The ALP assay was performed with culture supernatant samples (5 µL per lattice) every four days and on Day 14 with an ALP assay kit (Model ab83371; Abcam, Cambridge, UK) following the manufacturer’s instructions.

#### 2.3.3. Cell Count

On the last day of each experiment (Day 14), a manual cell count was performed. The media was removed, and the lattices were rinsed twice with 1.5 mL of PBS, 1.5 mL of Trypsin-EDTA (0.25%) was then added to each well and pipetted multiple times to facilitate the flow of Trypsin into the lattices. Cell count was manually performed with a hemocytometer containing 10 µL of cell suspension with an inverted microscope. The Trypsin in each well was then replaced, and a second cell count was performed. The final cell count was then calculated as the average of the two measurements.

## 3. Results

### 3.1. Glucose Concentration in Cell Culture Media Surrounding Titanium Lattices

**Figure 5:**
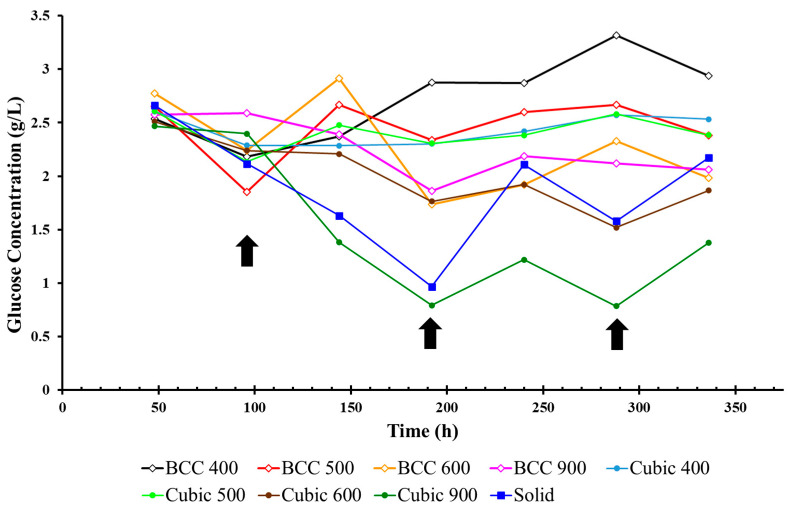
Glucose concentration for cubic and BCC lattices (arrows indicate when media was replaced).

The glucose concentration in the cell culture media surrounding porous Ti-6Al-4V lattice structures was measured every two days to estimate cellular metabolic activity over the duration of each experiment, as illustrated below for cubic and body-centered cubic (Figure 5) unit cell geometries, respectively (average across three experiments). The average glucose concentration over the duration of the experiment per pore size and unit cell geometry (average across three experiments) can be found in Table 1.

The greatest change in glucose concentration was observed for the Cubic 900 µm specimen between Day 4 (2.39 g/L) and Day 8 (0.79 g/L) of the experiment. This infers that more glucose was consumed and converted to adenosine triphosphate (ATP) by developing MG-63 cells that permeated the larger pores of Cubic 900 µm when compared to other specimens. The cubic specimens also demonstrated the greatest glucose consumption overall when compared to specimens with BCC unit cell geometries with the same pore sizes.

### 3.2. ALP Activity in Cell Culture Media Surrounding Titanium Lattices

**Figure 6:**
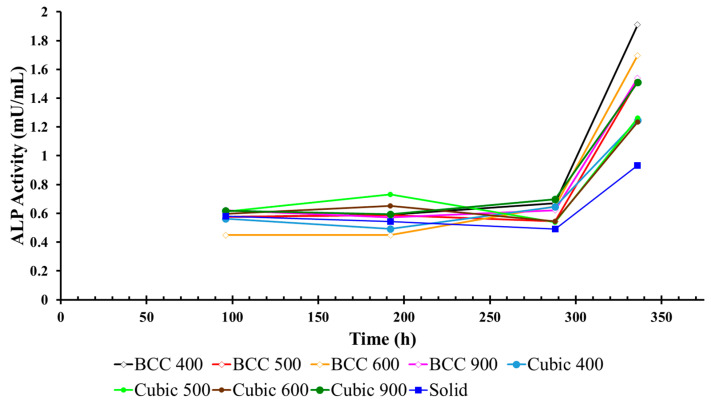
ALP activity in media surrounding BCC and cubic lattices.

ALP activity was measured every four days to evaluate osteoblast differentiation in the cell culture environment in between media changes, as illustrated below in Figure 6 for titanium specimens with cubic and BCC unit cell geometries (average across three experiments). The greatest change in ALP activity was observed during Days 12–14 for all cubic specimens, with the largest change measured in the media surrounding Cubic 900 µm specimens between Day 4 (0.61 mU/mL) and Day 14 (1.50 mU/mL) of the experiment (Table 2).

Similar to the results for cubic specimens, the greatest change in ALP activity was observed during Days 12–14 for all BCC specimens, with the largest change measured in the media surrounding BCC 400 µm specimens on the last day of the experiment (Table 2). However, due to the high standard deviation observed in BCC specimens, the results indicated that, by comparison, lattices with the cubic unit cell geometry outperformed their BCC counterparts with respect to osteogenesis, as indicated through the greatest change in ALP activity. When compared with glucose consumption data (Table 1), the ALP activity results may infer that Cubic 900 µm specimens promoted the greatest maturation of viable MG-63 cells from the formation (glucose consumption) to the mineralization phase of bone remodeling that is critical for osseointegration in metallic implants.

### 3.3. Structural Characterization of Cubic and Body-Centered Cubic Titanium Lattices

**Figure 7:**
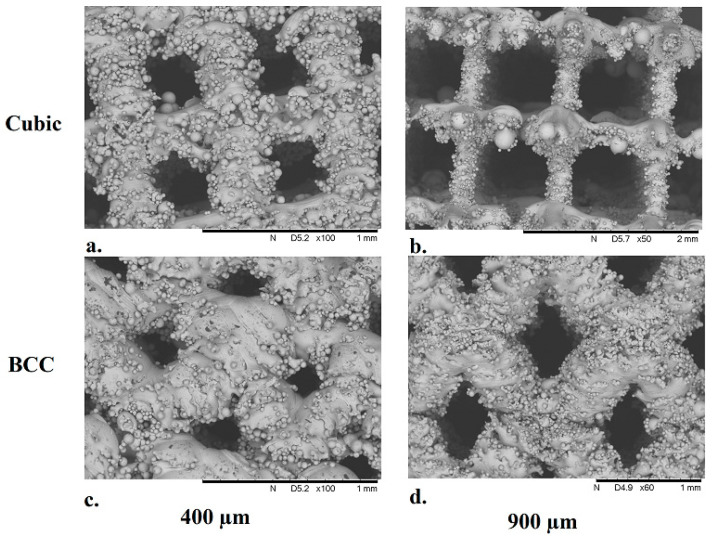
SEM images of 10 mm Ti-6Al-4V lattice specimens with BCC (**top row**) and cubic (**bottom row**) unit cell geometries. Pore sizes: 400 µm (**a**) and (**c**) and 900 µm (**b**) and (**d**).

Structural characterization of the additively manufactured Ti-6Al-4V cubic and BCC lattice specimens was performed using scanning electron microscopy (Hitachi SEM Model TM3000), as illustrated in the SEM images shown in Figure 7.

The average pore size and strut thickness were measured from the SEM images for each lattice specimen used in the experiments (Table 3) in order to assess variations in 3D printed parts across different SLM build jobs. Deviations in pore size and strut diameter measured across specimens when compared to the specified value in the associated CAD model indicate that additively manufactured lattices with cubic unit cell geometry produce more consistent results. The results also indicate that cubic lattices feature a larger pore size and smaller strut diameter when compared to their BCC counterparts, which may have facilitated an increase in glucose consumption by MG-63 cells that permeated into the lattice structures.

### 3.4. Analysis of Solid Titanium Specimen

**Figure 8:**
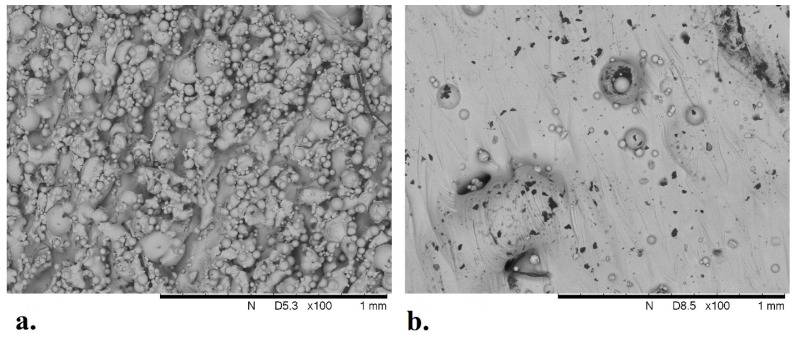
SEM image of a solid Ti-6Al-4V specimen, side profile (**a**) and top profile (**b**).

SEM images were taken of a solid Ti-6Al-4V specimen used in the experiments to analyze the surface roughness associated with the melt pool thermodynamics of the SLM process. As illustrated in Figure 8, there are numerous peaks and valleys in the surface finish that provide potential attachment sites for MG-63 cells in the culture media. The average size of unmelted Ti-6Al-4V powder particles found on the surfaces of the solid specimen was 34 µm for the sides of the specimen and 56 µm for the top surface. When compared with glucose concentration measurements (Figure 5), the data indicate an increase in cellular metabolic activity over time for the solid specimen (average across replicate experiments).

### 3.5. Glucose Concentration Per Available Surface Area of Titanium Lattices

**Figure 9:**
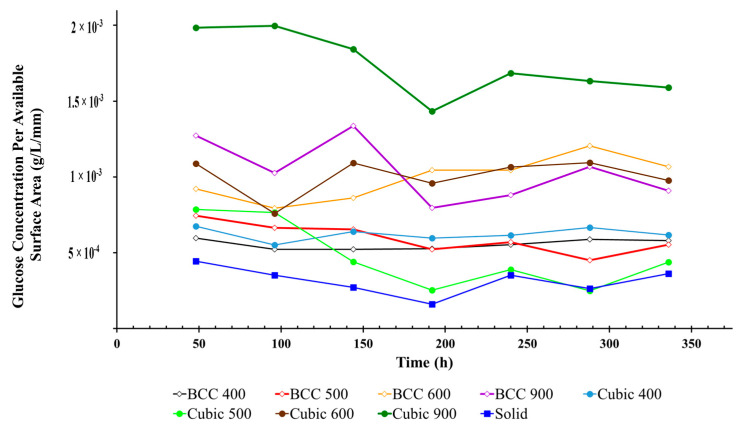
Glucose concentration per available surface area for BCC and cubic lattices.

As illustrated in Figure 7 and Figure 8, the additive manufacturing process results in surface roughness in the finished part due to a number of variables associated with SLM build parameters. Due to varying unit cell geometry and porosity, each lattice specimen also exhibits differences in the available surface area that can facilitate cell adherence required for osseointegration. In order to account for these variations and estimate the ‘efficiency’ of cell proliferation within the pores of lattice specimens, glucose concentration per available surface area was analyzed over time, as illustrated in Figure 9. For both structural geometries, it was found that the average glucose concentration decreased proportionally with pore size over the duration of the experiment. In addition, for both strain geometries, the lattice specimen with the largest internal pore size (900 µm) demonstrated the greatest glucose consumption. Across all experiments, the glucose concentration per available surface area was the lowest for the solid specimen, indicating that MG-63 cells permeated the pores of the other lattice specimens.

### 3.6. Cell Count in Media Surrounding Titanium Lattices

At the end of each experiment, the number of MG-63 cells was counted as a numerical method to estimate cell proliferation throughout the experiment (Table 4). In all cases, the lattices with cubic unit cell geometry exhibited higher cell counts when compared to their BCC counterparts with the same pore size. The highest cell count was measured in the media surrounding the lattices with the largest pore size of 900 µm for both cubic (1.90 × 10^6^ ± 6.92 × 10^5^ cells/mL) and BCC (900 µm: 8.33 × 10^5^ ± 2.65 × 10^5^ cells/mL) unit cell geometries. The highest cell count across all specimens was measured for Cubic 900 µm, followed by the solid specimen (1.84 × 10^6^ cells/mL).

## 4. Discussion

The purpose of this study was to determine which unit cell geometry and porosity promoted the highest level of osteogenic growth in MG-63 osteoblast-like cells amongst various additively manufactured biomimetic Ti-6Al-4V lattice structures. While it was found that all specimens were capable of facilitating osseointegration in vitro, the lattice structures with cubic unit cell geometries outperformed their BCC counterparts for all pore sizes. The greatest rate of change in glucose consumption was observed in Cubic 900 µm specimens between Days 4–8 of each experiment (Figure 5). When compared with ALP activity (Figure 6), which increased across all specimens during Days 12–14 of each experiment, the results indicated that Cubic 900 µm specimens promoted the maturation of viable MG-63 cells from the formation (surface attachment) to the mineralization phase of bone remodeling [30,31]. In addition, the highest cell count (Table 4) was measured for Cubic 900 µm specimens on the last day of the experiment, further exemplifying the biocompatibility of this particular titanium lattice structure for osseointegration in vitro.

In general, lattice specimens with the largest pore size (900 µm) facilitated the highest level of osteogenic growth when compared to their counterparts with smaller pore sizes, although BCC 900 µm exhibited a lower cell count than the solid specimen (Table 4). The literature has shown that larger pore sizes are ideal for cell proliferation and growth, whereas smaller pores can limit nutrient and waste exchange [10]. Smaller pore sizes can occlude bridging cells and reduce cell migration due to limited permeability [32], which may explain the poor performance of the 400 µm and 500 µm lattices in this research study. Bael, et al. [10] found that larger pores provided more open space for cells to grow and that the higher medium diffusivity from larger pores resulted in an increase in oxygen and nutrient supply.

Difficulties associated with 3D printing titanium lattices with smaller pore sizes were also observed in this study, in particular for specimens with BCC unit cell geometry. Due to current limitations in print resolution capabilities for state-of-the-art SLM technology, deviations in pore size and strut diameter were measured in the finished parts. The most profound deviations were observed in BCC 400 µm specimens (Table 3) when compared to the specified pore size in the corresponding CAD diagrams. These discrepancies in pore size and strut diameter are due to un-melted particles that attach to the surface of the lattices during the SLM process, as seen in the SEM images (Figure 7). In addition, it has been found that struts manufactured at an inclination (BCC lattices) will result in larger than designed struts due to overhang, which leads to additional bonded metal particles on the struts [33].

The BCC unit cell can be characterized as a cubic unit cell that is rotated 90°, most notably resulting in changes to mechanical properties [34]. However, this rotation may also facilitate a variation in the cell bridging phenomenon when compared to cubic lattices. Similar studies on metal lattices have indicated the occurrence of cell bridging. These cellular nanotubes [35] provide a direct conduit for signal propagation and the transfer of nutrients in and out of lattices, ultimately affecting glucose consumption, ALP activity, and cell viability [36]. Hollander, et al. [37] created Ti-6Al-4V lattices with cylindrical pores of 500, 700, and 1000 µm pore sizes, which were cultured with human primary osteoblasts. Live/dead staining revealed the 500 µm lattices had overgrown pores, whereas those with 700 and 1000 µm pores were not overgrown but rather exhibited circular cell growth on the rim of the pores. Bael, et al. [10] observed the occlusion of pores with cells by the bridging of corners in multiple unit cell geometries with pore sizes 500 µm and 1000 µm.

In addition to the lattice specimens with varying unit cell geometry and pore size, a solid specimen was included in the experiments of this study for comparison. The glucose concentration in the media surrounding the solid specimen decreased the most between Day 2 (2.66 g/L) and Day 8 (0.96 g/L) of the experiments, which is similar to the results for Cubic 900 (Table 1). However, the glucose consumption dropped more rapidly for the solid specimen during the same period of time (Figure 9). This may be due to increased surface area on the solid specimen (606 mm^2^) for cells in suspension to attach and proliferate, as validated by an increase in cell count when compared to all specimens other than Cubic 900 µm (Table 4). These results indicate that on average, more cells permeated and attached to surfaces within the pores of the Cubic 900 µm lattice structures than the (more proximal) exterior surface of the solid specimens. Surface area can be seen in Table 3 for all the lattice structures.

The sides of the solid specimen have a rougher surface than the top of the specimen (Figure 8), which is an inherent characteristic of the SLM process as unmelted powder particles attach to the side of an object during production. The cell count method used in this study relies on Trypsin to facilitate the release of the cells from the surfaces. While this process is straight-forward for the six-sided solid specimen, it is significantly more complicated for lattices with thousands of facets.

### Study Limitations

As discussed in prior sections, limitations in the SLM manufacturing process may result in inconsistencies in the printed part. In this study, variations in pore size and strut diameter were observed across lattice specimens when compared to the original CAD drawings. This was particularly true for lattices with body-centered cubic unit cell geometries. In future studies, this deviation in print resolution should be considered to achieve higher fidelity in additively manufactured parts.

In addition, at the end of the experiment, cell counts were only measured on the final day of the experiment due to limitations in the cell count procedure implemented, which effectively terminated the MG-63 cells. The additional cell count data may have allowed for a more in-depth comparison with other assays, including information on how the cells proliferate throughout the culture. Furthermore, this experiment may have benefited from a longer cell culture period. The ALP results (Figure 6) indicate a large spike during Days 12–14 at the end of the experiment. Other studies have found MG-63 cells can survive cultures up to twenty-eight days [38]. Finally, the standard deviation for some experiments was considerably large. With respect to the cell count data, the increase in standard deviation may be due to variances associated with manually counting cells instead of using an automated method. The results of the glucose assay (Table 1) also displayed an increase in standard deviation, which can be attributed to a small sample size of three experiments in addition to variances in assay performance.

## 5. Conclusions

In this work, we investigated the effects of porosity and unit cell geometry in SLM additively manufactured Ti-6Al-4V biomimetic lattice structures on the osteogenesis of human MG-63 osteoblastic cell lines in vitro, using glucose consumption, alkaline phosphatase (ALP) concentration, and end-of-culture cell count as markers for osteogenic growth. Two different strut geometries were utilized (cubic and body-centered cubic), along with four different pore sizes (400, 500, 600, and 900 µm, representing 40–90% porosity in a 10 mm cube), in addition to a solid specimen for comparison. The results indicated that pore size 900 µm had the highest glucose consumption, the largest glucose consumption per available surface area, the greatest change in ALP activity, and the highest cell count when compared to other pore sizes. In terms of unit cell geometry, cubic lattices, on average, exhibited higher glucose consumption, greater change in ALP activity (when standard deviation is considered), and a larger cell count when compared to their counterparts with BCC strut geometries. It can be concluded that Cubic 900 µm lattice structures outperformed other specimens in facilitating the maturation of viable MG-63 cells from the formation (surface attachment) to the mineralization phase of bone remodeling (as seen in ALP activity), offering the most promise for osseointegration in additively manufactured titanium implants in the future. However, irrespective of a particular pore size or unit cell geometry, it was found that all of the lattices were capable of promoting osteogenic growth. This was due, in part, to surface roughness in the finished parts, which is inherent of the additive manufacturing process. In addition, it was discovered that the SLM process created BCC lattices with reduced pore sizes and wider strut diameters, which negatively affected in vitro performance.

## Figures and Tables

**Table 1:** Glucose concentration (g/L) over time per pore size and strut geometry with standard deviation.

Pore Size (µm)	Strut Geometry	Glucose Concentration (g/L)							Standard Deviation (g/L)
		Day 2	Day 4	Day 6	Day 8	Day 10	Day 12	Day 14	
0	Solid	2.66 ± 0.28	2.11 ± 0.65	1.63 ± 0.38	0.96 ± 0.16	2.10 ± 0.58	1.57 ± 0.57	2.17 ± 0.53	0.45
400	Cubic	2.60 ± 0.19	2.28 ± 0.56	2.28 ± 0.51	2.30 ± 0.99	2.41 ± 0.70	2.57 ± 1.39	2.53 ± 1.10	0.78
	BCC	2.53 ± 0.13	2.18 ± 0.47	2.37 ± 0.81	2.87 ± 0.11	2.87 ± 0.13	3.31 ± 0.7	2.93 ± 0.03	0.35
500	Cubic	2.60 ± 0.11	2.13 ± 0.50	2.47 ± 0.38	2.30 ± 1.05	2.38 ± 0.79	2.58 ± 1.37	2.38 ± 1.49	0.81
	BCC	2.65 ± 0.19	1.85 ± 0.87	2.66 ± 0.47	2.33 ± 1.01	2.59 ± 0.92	2.66 ± 1.50	2.38 ± 0.83	0.83
600	Cubic	2.50 ± 0.19	2.23 ± 0.48	2.20 ± 0.50	1.76 ± 0.73	1.92 ± 0.72	1.51 ± 0.73	1.86 ± 0.66	0.57
	BCC	2.77 ± 0.38	2.23 ± 0.48	2.91 ± 0.34	1.73 ± 1.28	1.91 ± 1.31	2.32 ± 1.69	1.98 ± 1.38	0.98
900	Cubic	2.46 ± 0.14	2.39 ± 0.58	1.38 ± 0.22	0.79 ± 0.08	1.21 ± 0.10	0.78 ± 0.09	1.37 ± 0.14	0.19
	BCC	2.57 ± 0.18	2.59 ± 0.36	2.39 ± 0.34	1.86 ± 0.77	2.18 ± 0.78	2.11 ± 1.35	2.06 ± 0.78	0.65

**Table 2:** Average ALP activity in media surrounding cubic and BCC lattices with standard deviation.

Pore Size (µm)	Strut Geometry	ALP Activity in Media (mU/mL)				Standard Deviation (mU/mL)
		Day 4	Day 8	Day 12	Day 14	
0	Solid	0.57 ± 0.37	0.54 ± 0.30	0.48 ± 0.10	0.93 ± 0.26	0.17
400	Cubic	0.56 ± 0.17	0.49 ± 0.24	0.64 ± 0.26	1.24 ± 0.45	0.29
	BCC	0.57 ± 0.24	0.59 ± 0.29	0.66 ± 0.25	1.90 ± 0.96	0.56
500	Cubic	0.61 ± 0.12	0.73 ± 0.58	0.53 ± 0.17	1.26 ± 0.50	0.28
	BCC	0.57 ± 0.20	0.58 ± 0.40	0.54 ± 0.21	1.52 ± 0.75	0.41
600	Cubic	0.59 ± 0.22	0.65 ± 0.37	0.54 ± 0.16	1.23 ± 0.48	0.27
	BCC	0.44 ± 0.26	0.44 ± 0.27	0.64 ± 0.35	1.69 ± 0.84	0.51
900	Cubic	0.61 ± 0.26	0.59 ± 0.38	0.69 ± 0.27	1.50 ± 0.72	0.38
	BCC	0.62 ± 0.21	0.57 ± 0.32	0.62 ± 0.24	1.53 ± 0.68	0.40

**Table 3:** Measurements and deviations in Ti-6Al-4V lattice pore size and strut diameter obtained from SEM images. Surface area is estimated in CAD.

Pore Size (µm)	Strut Geometry	Averaged Measured Pore Size (µm)	Pore Size Deviation (%)	Averaged Measured Strut Diameter (µm)	Strut Diameter Deviation (%)	Surface Area (mm^2^)
400	Cubic	374.6	−6.5	310.1	55.0	3671
	BCC	219.2	−45.2	428.5	114.2	4181
500	Cubic	439.2	−12.1	333.8	66.9	2982
	BCC	411.5	−17.7	344.8	72.4	3166
600	Cubic	559.1	−6.8	268.2	34.1	2330
	BCC	533.6	−11.0	375.5	87.6	2655
900	Cubic	849.5	−5.7	312.6	56.3	1262
	BCC	675.2	−24.9	629.16	57.2	2167

**Table 4:** Cell count for cubic and BCC lattices on Day 14 (average across three experiments), with percent differences between cubic and BCC.

Pore Size (µm)	Strut Geometry	Average Estimated Cell Count (Cells/mL)	Standard Deviation (Cells/mL)	Percent Difference (%)
0	Solid	1.84 × 10^6^	7.62 × 10^5^	-
400	Cubic	9.93 × 10^5^	5.42 × 10^5^	75.2
	BCC	4.50 × 10^5^	8.29 × 10^4^	
500	Cubic	1.15 × 10^6^	6.04 × 10^5^	34.4
	BCC	8.12 × 10^5^	5.79 × 10^5^	
600	Cubic	8.33 × 10^5^	2.33 × 10^5^	112.5
	BCC	2.23 × 10^5^	1.67 × 10^5^	
900	Cubic	1.90 × 10^6^	6.92 × 10^5^	78.1
	BCC	8.33 × 10^5^	2.65 × 10^5^	

## Data Availability

The original contributions presented in the study are included in the article; further inquiries can be directed to the corresponding author.
